# Use of Flubendazole and Fenbendazole for Treatment of Lung Severe Infection by the Gapeworm *Cyathostoma bronchialis* (Nematoda: Syngamidae) in *Branta hutchinsii*, *Anser indicus* and *B. leucopsis* Exotic Geese: An Interesting Case

**DOI:** 10.3390/vetsci8080147

**Published:** 2021-07-29

**Authors:** Alessandro Guerrini, Andrea Carminati, Laura Stancampiano, Paola Roncada, Matteo Frasnelli

**Affiliations:** 1 Department of Veterinary Medical Sciences, University of Bologna, Via Tolara di Sopra 50, 40064 Ozzano dell’Emilia (Bologna), Italy; laura.stancampiano@unibo.it (L.S.); paola.roncada@unibo.it (P.R.); 2 Department of Veterinary Sciences, University of Turin, Via Largo Paolo Braccini 2, 10095 Grugliasco (Turin), Italy; andrea.carminati@edu.unito.it; 3 Istituto Zooprofilattico Sperimentale della Lombardia e dell’Emilia Romagna “Bruno Ubertini”, Sede Territoriale di Ravenna, Via del Limite 2, 48022 Lugo (Ravenna), Italy; matteo.frasnelli@izsler.it

**Keywords:** *Cyathostoma bronchialis*, geese, flubendazole, fenbendazole

## Abstract

A 6-year-old female goose (*Branta hutchinsii*) from a group of ornamental exotic geese was found dead due to severe respiratory failure, followed by emission of haemorrhagic sputum and blood clots from the beak and nostrils, and then collapse. At necropsy, the cause of death was attributed to a total of 76 helminth parasites found in the trachea and lungs, then identified as *Cyathostoma bronchialis*. The flock was initially treated by feed with flubendazole (1200 g/1000 kg/feed) for 7 consecutive days but, at the reappearance of the respiratory symptoms 10 days after, the animals were given fenbendazole by drinking water (300 mg/L) for 7 consecutive days, but at the reappearance of the respiratory symptoms 10 days after, the animals were given fenbendazole via drinking water (300 mg/L) for 7 consecutive days. Despite these treatments, the respiratory symptoms continued to relapse 10–15 days after the end of drug administration. In the literature, there are no data regarding drugs for the treatment of *C. bronchialis* infestations in geese, and the use of these drugs in exotic birds occurs as “*off-label*” use. This case study provides information on *C. bronchialis* life cycle, which is still poorly studied and poorly documented today. In particular, the case provides useful suggestions for evaluating an appropriate protocol for the treatment of *C. bronchialis* in geese.

## 1. Introduction

The sub-family *Syngaminae* (Order Strongylida) comprises parasites of respiratory tracts of mammals (genera *Mammomonogamus* and *Rodentogamus*) and birds (genera *Boydinema* spp., *Cyathostoma* spp., *Syngamus* spp.), the latter commonly known as gapeworms [1]. The life cycles of these parasites are peculiar and differ from each other even though they belong to the same family. Gapeworms are parasites located in the trachea, bronchi and lungs of land and waterbirds, and most of the known species are in the typical Y-shape and attach to the tracheal mucosa. The largest is the genus *Cyathostoma*. This genus was established by Blanchard and Threlfall for *Cyathostoma lari*, species described after being found from the orbital cavity of the black-headed gull (*Chroicephalus ridibundus*) [2,3]. In the most recent taxonomic system of *Syngaminae* proposed by Lichtenfels [4], the genus *Cyathostoma* is divided into two sub-genera in relation to the structure of copulatory bursa and spiculae length: *Cyathostoma* (*Cyathostoma*) in which the dorsal ray extends beyond the end of the copulatory bursa to form characteristic thorn-like projections, and *Cyathostoma* (*Hovorkonema*) [2,3,4,5], in which the dorsal ray does not extend beyond the end of the copulatory bursa. However, in the classification systems of *Syngamidae* created by Ryzhikov [6] and Baruš and Tenora [7], *Cyathostoma* and *Hovorkonema* are treated as independent genera. The occurrence of *Cyathostoma* nematodes has been reported in a variety of species of birds from several orders [1,7,8,9]. In particular, Ali [10] reported cyathostomiasis cases from the nasal or infraorbital cavities or the trachea of at least 10 bird families but mainly in waterbirds. Although in the past the taxonomic of the species have been discussed [10,11,12], recently, Kanarek et al. recognized *C.* (*Hovorkonema*) *bronchialis* as a valid species [1]. Adult worms are localized in the larynx, trachea, bronchi and sporadically in abdominal air sacs. The adult form of *C. bronchialis* is similar to *Syngamus* spp. but not strictly in copula. The final host becomes infected by directly ingesting the larvae of *C. bronchialis*, but chances of infection increase in the case of ingesting a paratenic host, such as earthworms. Third-stage larvae of *C*. *bronchialis* migrates to the lungs through the peritoneal cavity and air sacs rather than the blood stream, as *S. trachea* does. The final moult of fourth-stage larvae take place in the lungs. After *C*. *bronchialis* migrates into the trachea at 6 days and copulates by 7 days post-infection (p.i.), the eggs can be found 13 days p.i. in the tracheal mucus. Infected birds present dyspnoea, air gasping and emit a grunting sound due to the difficulty in breathing caused by parasites and by an excess of mucus as a result of irritation of the tracheal lining. The death occurs from suffocation caused by the worms that can easily obstruct the trachea and bronchi with severe pneumonia [13]. A hyperplasia of the bronchi’s epithelium during the prepatency phase due to the ingestion of the nematode’s eggs is also possible [14]. *Syngamus* spp. are cosmopolitan parasites, typical of *Galliformes* and *Passeriformes* but recorded also in other bird species, such as *Anseriformes*, *Ardeiformes*, *Pelecaniformes*, *Otidiformes*, *Piciformes* and *Cypseliformes* [15]. *S. trachea* usually causes a huge economic loss in the poultry industry, where outbreaks have caused a substantial loss, especially in young turkeys, guinea fowls or pheasants, which are all highly susceptible to this particular type of nematode [16]. As already stated, the adult form of the parasite is located in the trachea, bronchi and bronchioles in a typical Y-shaped form [17]. Adult birds rarely reveal signs of infection, except in cases of massive infections [18]. The reproductive cycle of *S. trachea* may include one paratenic host in addition to the final avian host [19], such as earthworms, snails or insects (*Eisenia foetidus*, *Allolobophora caliginosus*) [20,21]. After ingestion, some larvae penetrate through the oesophagus and migrate directly to the lungs or trachea; however, the majority of the ingested larvae make their way to the duodenum and, after passing through the portal blood stream, enter the lungs via the liver portal system and heart. Larvae are found in the liver 2-h p.i. and in lungs 4-h p.i. The worms in copula can be found 5 days post-infection in pheasant, but in chickens, a pair of worms can be seen a day later [22]. The prepatent period is 12–17 days in chickens, and the adults can live 2–4 months.

Usually, the treatment of these parasites consists in the use of benzimidazoles, which have larvicidal and adulticidal effects. A number of anthelminthic compounds are available for the control of nematodes in food-producing animals; however, the options are limited in avian species, particularly in geese. In Italy, the lack of authorization for the use of some anti-parasitic drugs in poultry, in particular in ornamental poultry, which is not recognized by the Italian legislation as a pet, places serious limits on the treatment of some parasitosis. Furthermore, the parasitosis in domestic birds are scarcely investigated, especially in ornamental poultry. This paper describes an interesting case of pulmonary parasitosis in a group of ornamental exotic geese and the effects of pharmacological treatment with flubendazole and fenbendazole against *C. bronchialis* infection.

## 2. Materials and Methods

### 2.1. Farm and Flock

**Figure 1:**
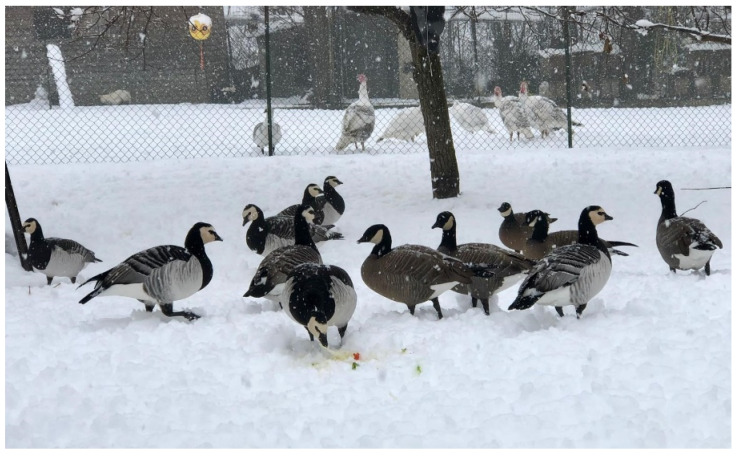
Group of Minor Canadian geese (*Branta hutchinsii*), Barred head geese (*Anser indicus*) and White-faced geese (*Brenta leucopsis*).

The sampling farm is located on the Piedmont hills in the open countryside with an expansion of 5000 square meters. The group of geese considered in the study was composed of 3 males and 3 females (3 couples) of *Branta hutchinsii*, all aged between 3 and 7 years, raised in the same aviary cage with 2 couples of *Anser indicus* and 4 couples of *B. leucopsis* in a free-range system (Figure 1). The other waterfowl species were raised separately in another aviary cage, divided by species or groups of species, compatible in character and reproductive period. In other aviary cages, different poultry breeds were rared. Each group of animals had a pond at his disposal, where the water was changed every 30 days, without added chlorine, and meadow for scratching. The reproduction occurs naturally between April and May, followed by the hatching, and each couple takes care of their goslings independently. The average spring, summer, autumn and winter temperatures ranged around +8/+15 °C, +28/+35 °C, +5/+20 °C and 0/−20 °C, respectively.

### 2.2. Diet

The group of geese was fed with a mixed cereals diet (corn, wheat, sorghum, soy flour) and a commercial pelleted feed specific for exotic geese. The composition of the pelleted feed is shown in Table 1. During winter, the proteins and fat values were increased by about 17.5% and 5%, respectively. Feed and water were provided *ad libitum*. Daily, vegetables and fruits were offered in addition to the internal pasture consisting of polyphite lawn, and periodically, the farmer used to provide multivitamin supplements in drinking water, particularly during the reproduction period.

### 2.3. Periodic Pharmacological Treatments on the Farm

The animals were not subject to constant or preventive drug treatments. The only treatments were against helminths if present and confirmed by parasitological exam, with a commercially medicated premix of flubendazole 50 mg/g (600 g/1000 kg/feed, 30 mg/kg), administered for 7 consecutive days and repeated after 20 days for 7 consecutive days, as indicated by the manufacturer’s recommendation. A coprological exam was normally performed to verify the efficacy of the treatment. Flubendazole was the only drug used by the farmer for the treatment of the animals. There was no alternation with other drugs in the 3 years prior to this study, and the last treatment was performed 32 days before the clinical case.

## 3. Case History and Anamnestic Data

### 3.1. Acute Respiratory Syndrome: First Occurrence

Between March and April 2019, the group of geese presented different respiratory symptoms among the subjects. The symptomatology appeared more severe in females of *B. hutchinsii* with stronger sneezing followed by an air-hunger attitude for a few seconds than males of the same species. In couples of *B. leucopsis*, the symptomatology was milder, with only frequent sneezing and light shaking of the head, while in *A. indicus* couples were totally asymptomatic. The animals were treated with enrofloxacin 5%, for 7 days at a dose of 0.5 mL/kg body weight (BW) by individual intramuscular injection (i.i.), for suspected bacterial pneumonia. The farmer reported that the respiratory symptoms had apparently resolved after 7 days of treatment and that the month before the start of clinical symptoms, the coprological exam, performed on a pool of faeces from the group of geese, was positive only for nematode eggs. Therefore, the animals were treated, as previously reported, 32 days before. Since the antibiotic treatment in mid-May, no more anthelmintic treatments were administered. In mid-May, during the hatching period, the same symptomatology reappeared in the group, more severe only in the *B. hutchinsii* females, with more frequent sneezing, air-hunger attitude, gurgling and head shaking with consequent acute respiratory distress. No enteric or nervous symptoms were reported. The farmer reported also that respiratory symptoms appear only during the spring–summer season and not during autumn–winter. A 6-year-old female goose (*Branta hutchinsii*) in hatching was found dead due to severe respiratory failure, followed by emission of haemorrhagic sputum and blood clots from the beak and nostrils, and then collapse. Another female died after few days with the same symptomatology, but only one carcass was delivered frozen to the laboratory of the Istituto Zooprofilattico Sperimentale della Lombardia e dell’ Emilia Romagna, a section of Lugo, Ravenna (Italy) for more in-depth diagnoses.

### 3.2. Necropsy Observations and Parasites Indentification

**Figure 2:**
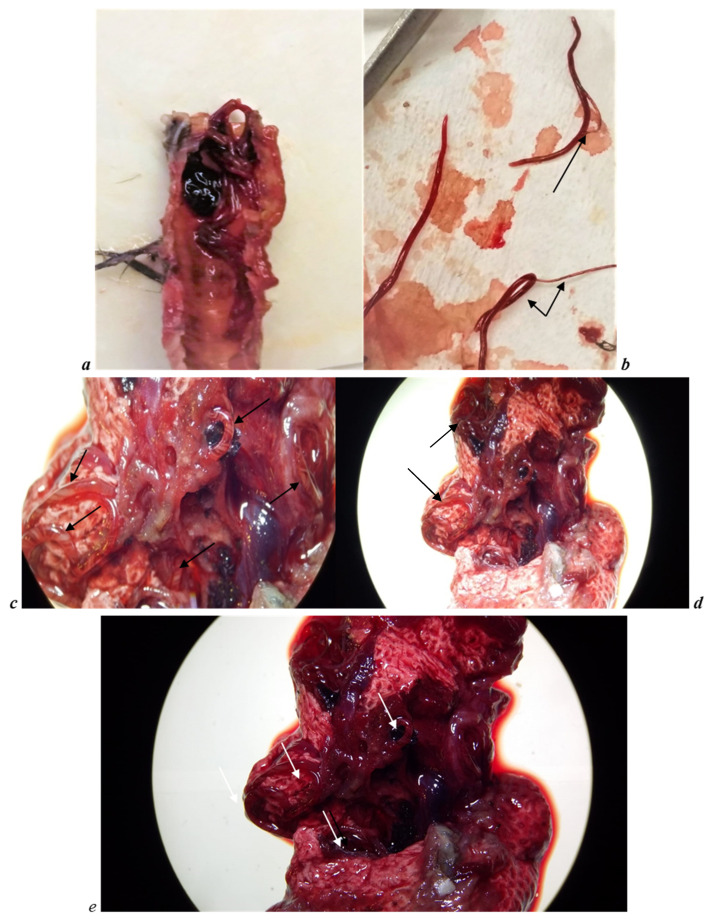
(**a**) Tracheal lumen with nematodes and blood clots. (**b**) Nematodes in the Y-shape form (copula), as shown in the figure by the arrows. (**c**–**e**) Pulmonary oedema and haemorrhagic flooding with the presence of numerous nematodes, as shown in the figure by the arrows.

Only the 6-year-old female of *B. hutchinsii*, dead as previously described, was examined. From the necropsy observations, the goose was in normal body condition considering the breeding season. Feathers were in a good state, and no faecal soiling in the cloacal area was observed. From an exterior approach, some blood clots were revealed inside the beak and nostrils, with a catarrhal haemorrhagic exudate. At dissection, there was catarrhal haemorrhagic exudate in the oropharynx, present throughout the tracheal lumen up to the tracheobronchial bifurcation (Figure 2a). In the trachea, there were few parasitic forms referable to nematodes (Figure 2a). Twenty-nine of these (61.70%) were in Y-shaped form (Figure 2b), but the remaining parasites (*n* = 18, 38.29%) were single and not in a couple, for a total of 76 adult parasites. There were pulmonary edema and haemorrhagic flooding with the presence of numerous nematodes referable to nematodes of the Family *Syngamidae* (Figure 2c–e). Other observations that emerged during necropsy were hepatomegaly and renal congestion. The intestine was dilated with the presence of enteritis and catarrhal serum typhlitis but without the presence of any helminth parasites. At the Department of Veterinary Medicine (Bologna, Italy), parasites identification was conducted. Adult parasites were isolated from the trachea of the dead goose, stored in alcohol 70%, clarified in lactophenol and morphologically identified thanks to the keys and descriptions of Chapin, Cram, McDonald, Fernando et al. and Anderson et al. [13,23,24,25,26].

## 4. Results

### 4.1. Parasites Identification

**Figure 3:**
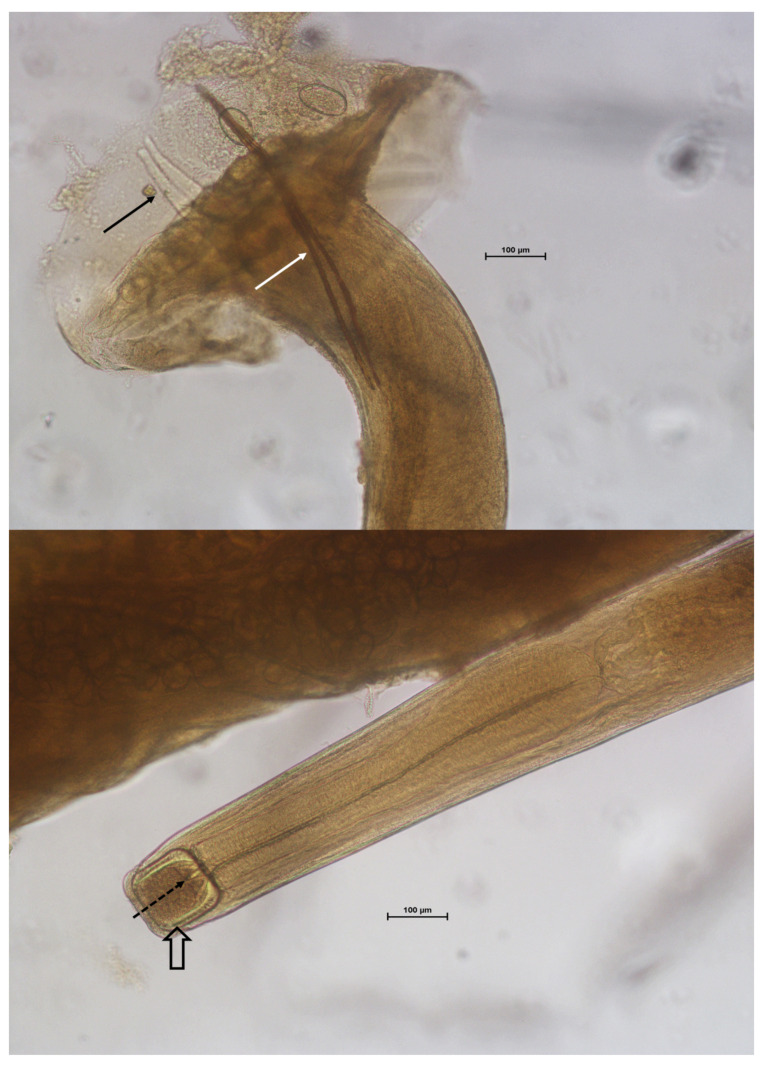
Microscopic view of the tail and head of *C. bronchialis* male. Black arrow: branched dorsal ray; white arrow: spicules; empty arrow: buccal capsule; dotted arrow: buccal teeth. Unit is 100 µm.

The parasites collected were identified as *C. bronchialis*. Besides tracheal localization, the main features useful for the identification were: males smaller than females; males and females not strictly joint in copula; buccal capsul that was large and heavily walled with teeth of different sizes at the base; male with strongyliform bursa with dorsal ray branched; spicules more than 400 μm long (range 620–680 μm) (Figure 3); and the female vulva near the anterior third of the body.

### 4.2. Follow Up

After the diagnosis of cyathostomiasis, all subjects of the 3 species, symptomatic and asymptomatic, were treated via feed with flubendazole at a dose of 1200 g/1000 kg/feed (60 mg/kg) for 7 consecutive days; a double dosage compared to that normally used in the feed and maximum dosage that can be used according to the manufacturer’s recommendation. The coprological exam was negative after treatment. After 48 h of treatment, the respiratory symptoms disappeared, yet they reappeared 10 days after the end of the treatment in the subjects of *B. hutchinsii* and *B. leucopsis*, while *A. indicus* remained asymptomatic. For this reason, the drug was changed for treatment and fenbendazole was used administered via drinking water at a dose of 300 mg/L (300 mg/kg) for 7 consecutive days. Medication was administered via drinking water, and access to the pond was prevented during the entire treatment period to ensure that medicated drinking water was the only disposable source. This treatment led to the remission of the respiratory symptoms after 24 h. The coprological exam was negative again after the treatment with fenbendazole, but respiratory symptoms reappeared after 15 days after the end of the treatment. The farmer reports that despite the treatment, the symptoms reappear seasonally in the months of April and May in the groups of geese, more severe in *B. hutchinsii*.

## 5. Discussion

### 5.1. Parasite Identification and Life-Cycle Considerations

*Cyathostoma bronchialis* is the typical gapeworm observed in wild and domestic waterfowl, consistently with parasite identification in the present study. Although its endogenous life cycle is reasonably well known in geese [13], little is known about its ecology, epidemiology and control. The observed clinical course of the disease suggests both environmental factors influencing its seasonal occurrence and different resistance or resilience to the infection related to the host species. The infection appears to endure notwithstanding repeated anthelmintic dosing, possibly because of the persistence of parasitic infective stages in paratenic hosts or because of the persistence of resistant larval stages in the definitive host. Being the prepatent period of about 7–13 days [13], the reappearance of the respiratory symptoms soon after 10–15 days after treatment will be possible, but probable as a unique cause of the observed pattern only if there is high environmental contamination and therefore a high probability of immediate reinfection, coupled with poor effective host immunity. Fernando and Barta report effective immunity to *Syngamus trachea*, but no data are available for *C. bronchialis* [27]. An alternative hypothesis should be formulated supposing that *C. bronchialis*, consistently with other well studied nematodes of the order Strongylida, could have a developmental arrest at the larval stage inside the definitive host [28,29] whose duration is prolonged by the presence of adult parasites that enhance host immunity. If this is the case, the treatment killing adult parasites would produce the observed paradoxical effect [30,31], allowing the rapid development of larval stages in adults.

### 5.2. Pharmacological Treatment and Considerations

The benzimidazoles, such as fenbendazole and flubendazole, have the same mode of action and are currently the only active substances licensed for use in laying hens and poultry (pheasants and turkeys) in the European Union countries [32]. In particular, various products marketed in Italy containing flubendazole or fenbendazole (i.e., Panacur AquaSol^®^, Intervet International B.V., AN Boxmeer, The Netherlands, Flubenvet^®^, Elanco GmbH, Bad Homburg, Germany, Gallifen^®^, Huvepharma NV, Sofia, Bulgaria, etc.) and authorized for the treatment of some parasites in poultry do not mention its use in the treatment of *C. bronchialis* infection. Therefore, the use of these drugs in the treatment of nematodes in exotic birds occurs as “*off-label*” use. In this clinical case, the drugs were used according to the procedures envisaged for the “*off-label*” use of the drug. On parasites, fenbendazole acts on the pre-adult (L5 stage) and adult stages, as in the case of *Ascaridia galli*, *Heterakis gallinarum* and *Capillaria* spp., on which flubendazole is also active and effective. Flubendazole is also used and authorized in poultry and game [33,34]. In the literature, specific pharmacological treatments for *C. bronchialis* are not available. For this reason, the specific treatment for *S. trachea* was rated as the best possible option also due to the similarity of the parasites, including not only flubendazole and fenbendazole but also mebendazole. According to the study conducted by Vanparijs on geese, flubendazole was administered at 30 mg/kg in feed for 7 consecutive days [35]. This dosage has been established to be a safe and highly active anthelmintic for geese. In a study conducted by Kirsch in pheasants and partridges, *S. trachea* was reduced by more than 90% by a 4-day administration of 100 mg/kg of fenbendazole in feed [36]. These results are in accordance with findings obtained by Enigk and Dey-Hazra [37,38]. In studies in turkeys artificially infected with *S. trachea*, an anthelmintic effect of more than 90% and 98% was observed, respectively, 7 and 15 days after the administration of fenbendazole in feed at a dose of 5 mg/kg BW for 3 consecutive days [39]. Another possible treatment was described by Enigk and Dey-Hazra [40] in an experiment including 32 chickens experimentally infected with *S. trachea*, where the mebendazole was given at 40 mg/kg BW, through feed, on 3 successive days. The mebendazole removed the immature and mature gapeworms completely. The anthelmintic effect of mebendazole was also tested on 18 geese naturally infected with nematodes *Amidostomum anseris*, *Trichostrongylus tenuis* and *Capillaria anatis* and cestodes *Hymenolepis lanceolata* and *Hymenolepis setigera*. The treatment removed the nematode infection completely at a dose of 10 mg/kg BW and the cestode infection at a dose of 30 mg/kg BW, for 3 consecutive days, through feed. Furthermore, in another study conducted on geese, animals were treated against *Hymenolepis* spp. using mebendazole via feed for 6 consecutive days, given at a dose of 10 mg/kg BW. This treatment removed the infection partly, but when geese were treated at a dose of 30 mg/kg BW, the treatment eliminated the whole worm burden [40].

In our study, according to the results of the coprological tests that were negative after the treatment, fenbendazole and flubendazole would seem to have an adulticidal effect. From a clinical-pharmacological standpoint, the treatment with a dosage of 30–60 mg/kg of flubendazole in feed or 300 mg/kg of fenbendazole via drinking water of the group of geese is effective against the intestinal parasite forms, but the efficacy remains questionable against pulmonary infections despite the disappearance of symptoms, as this is not indicative of the parasitosis resolution. Indeed, the reappearance of symptoms at 10- or 15-days post-treatment depending on the used drug could explain the elimination of the larvae present in the trachea and not the ones in the lungs, which after becoming adult, trigger the respiratory symptoms. However, both periods are consistent with the normal life cycle of *C. bronchialis*, but we do not know why there is a difference of 5 days in the reappearance of respiratory symptoms, based on the use of one or the other drug. A further observation attributable to the use of one or the other drug is the time post-treatment in which the symptoms regress, but it is not clear whether it depends on the degree of infestation or the bioavailability that changes between flubendazole and fenbendazole conditioned by drinking water administration versus feed, which would facilitate its absorption. The helminticidal effect of flubendazole could also be lower than expected, as it has always been used in medicated feed at minimal dosages for several years. This may also have caused a resistance from the parasite. In the literature, pharmacokinetic studies in geese also remain very scarce, if not non-existent. Some chemical and pharmacological properties of flubendazole and fenbendazole, such as poor solubility in water, a poor intestinal absorption and, consequently, a hypothetical faster intestinal and liver metabolism (first-pass effect), were demonstrated in different studies in laying hens [35,41]. Nonetheless, the overall very low serum concentrations observed in laying hens during ascarid infections treatment do not translate to very poor efficacy when acting on adult parasites in the intestine [32]. After absorption, the benzimidazoles are rapidly metabolized in the liver mainly into their sulfoxide (oxfendazole) and, subsequently, into sulphone (oxfendazole sulphone) by ketoreduction [33,34]. In chickens, oxfendazole sulphone is the main metabolite found in plasma. Fenbendazole and flubendazole and their metabolites are distributed throughout the body, reaching concentrations higher in the liver, and their elimination is mainly through faeces, but the drug concentrations that could be found in the lung are not known. The elimination is more rapid in turkey and pheasant. The metabolites retain the benzimidazole structure and have toxicological properties similar to those mother compounds [33,42]. For these considerations, we cannot exclude that the variability of species, the modality of drug administration and the standard therapeutic protocols normally used for other poultry species can affect the efficacy of the treatment of this parasitosis in exotic geese. Not least, it should also be considered that the natural intake of large quantities of water by the geese, as typical behaviour, with consequent washout of the intestinal tract and production of very watery faeces, presumably contributed to further poor intestinal absorption of the drug. Another hypothesis could be that the animals being in hatching season, in particular the dead goose, did not ingest enough medicated water, decreasing the therapeutic effect of the treatment further.

## 6. Conclusions

This case study shows how much these goose species and their parasites are still little studied today, especially from a pharmacological point of view. The breeding of ornamental and exotic poultry, which can present metabolic, physiological and sensitivity differences to some parasites among the different raised species, and the scarce possibility of using other than authorized drugs, requires further diagnostic and therapeutic investigations. In particular, it would be necessary to set up specific therapeutic protocols for the treatment of parasites, specifically for *C. bronchialis*. This is a little known and studied parasite that proves to have particularities in its life cycle in these animals living in open environments and in continuous contact with parasites and paratenic hosts, which directly influence their life cycle and effectiveness of treatments. In particular, the case provides useful suggestions for evaluating an appropriate protocol for the treatment of *C. bronchialis* in geese. This additional knowledge is also necessary for correct and prudent use of the drug, given that the use of some “*off-label*” drugs poses legislative problems for veterinarians.

## Figures and Tables

**Table 1:** The composition of the pellet feed specifically for exotic geese.

Components	Nutritional Values (%)	Additives (* IU/mg/kg/Feed)
Corn bran–Wheat bran–Wheat–Soy flour–Brewers yeast–Soybean oil–DL-Methionine–L-Lysine	* CP: 17.1%–* CF: 3.7%–* CFi: 4.8%–Ash: 4.5%–DL-Methionine: 1.500 mg–L-Lysine: 1.500 mg–Umidity: 12.5%	Vit. A: 9.950 IU–Vit. D_3_: 2.701 IU–Vit. E 38 mg–Vit.K_3_: 3.25 mg–Vit.B_1_: 3.25 mg–Vit. B_2_: 7.8 mg–Vit. B_6_: 5.2 mg–Vit. B_12_: 0.02 mg–Niacin: 39 mg–Ca-D-pantotenate: 10 mg–Folic acid: 0.65 mg–Biotin: 0.1 mg–Cu: 5 mg–Mn: 50 mg–Se: 0.13 mg–Zn: 50 mg

* CP: Crude Proteins; * CF: Crude Fats; * CFi: Crude Fibre, * IU: International Units.

## Data Availability

Not applicable.
